# Disrupted morphological grey matter networks in early-stage Parkinson’s disease

**DOI:** 10.1007/s00429-020-02200-9

**Published:** 2021-04-07

**Authors:** Xueling Suo, Du Lei, Nannan Li, Wenbin Li, Graham J. Kemp, John A. Sweeney, Rong Peng, Qiyong Gong

**Affiliations:** 1 Huaxi MR Research Center (HMRRC), Department of Radiology, West China Hospital of Sichuan University, No. 37 Guo Xue Xiang, Chengdu, 610041 PR China; 2 Department of Psychiatry and Behavioral Neuroscience, University of Cincinnati, Cincinnati, OH USA; 3 Department of Neurology, West China Hospital of Sichuan University, Chengdu, Sichuan China; 4 Liverpool Magnetic Resonance Imaging Centre (LiMRIC) and Institute of Life Course and Medical Sciences, University of Liverpool, Liverpool, UK; 5 Research Unit of Psychoradiology, Chinese Academy of Medical Sciences, Chengdu, Sichuan China; 6 Functional and Molecular Imaging Key Laboratory of Sichuan Province, West China Hospital of Sichuan University, Chengdu, Sichuan China

**Keywords:** Parkinson’s disease, Early-stage, MRI, Graph theory, Brain network, Psychoradiology

## Abstract

While previous structural-covariance studies have an advanced understanding of brain alterations in Parkinson's disease (PD), brain–behavior relationships have not been examined at the individual level. This study investigated the topological organization of grey matter (GM) networks, their relation to disease severity, and their potential imaging diagnostic value in PD. Fifty-four early-stage PD patients and 54 healthy controls (HC) underwent structural T1-weighted magnetic resonance imaging. GM networks were constructed by estimating interregional similarity in the distributions of regional GM volume using the Kullback–Leibler divergence measure. Results were analyzed using graph theory and network-based statistics (NBS), and the relationship to disease severity was assessed. Exploratory support vector machine analyses were conducted to discriminate PD patients from HC and different motor subtypes. Compared with HC, GM networks in PD showed a higher clustering coefficient (*P* = 0.014) and local efficiency (*P* = 0.014). Locally, nodal centralities in PD were lower in postcentral gyrus and temporal-occipital regions, and higher in right superior frontal gyrus and left putamen. NBS analysis revealed decreased morphological connections in the sensorimotor and default mode networks and increased connections in the salience and frontoparietal networks in PD. Connection matrices and graph-based metrics allowed single-subject classification of PD and HC with significant accuracy of 73.1 and 72.7%, respectively, while graph-based metrics allowed single-subject classification of tremor-dominant and akinetic–rigid motor subtypes with significant accuracy of 67.0%. The topological organization of GM networks was disrupted in early-stage PD in a way that suggests greater segregation of information processing. There is potential for application to early imaging diagnosis.

**Supplementary Information:**

The online version contains supplementary material available at 10.1007/s00429-020-02200-9.

## Introduction

Parkinson's disease (PD) is a progressive neurological disorder with a variety of motor and non-motor features (Jankovic 2008). The primary pathologic process involves disruptions in the nigrostriatal dopamine system, but neuroimaging has demonstrated functional and structural abnormalities in multiple brain regions (Weingarten et al. 2015; Atkinson-Clement et al. 2017; Pan et al. 2017; Ji et al. 2018; Suo et al. 2021). Integrated analysis of the whole brain network may provide a more comprehensive understanding of brain abnormalities in PD.

The connectome (Sporns et al. 2005) approach, which models brain anatomy and function using graph analysis, characterizes brain anatomy as a complex network of nodes and edges from which graph metrics can be calculated to describe network attributes (Craddock et al. 2013). The brain has 'small-world' organization (Watts and Strogatz 1998), which facilitates the efficient segregation and integration of complex networks with low energy and wiring costs (Rubinov and Sporns 2010; Liao et al. 2017). Using noninvasive neuroimaging, graph theoretical analysis has revealed PD-related disconnection patterns in both functional (Suo et al. 2017; Berman et al. 2016; Sang et al. 2015; Luo et al. 2015; Fang et al. 2017; Ma et al. 2017b; Gottlich et al. 2013; Vancea et al. 2019) and structural white matter networks (Galantucci et al. 2017; Shah et al. 2017; Kamagata et al. 2018; Tinaz et al. 2017; Aarabi et al. 2015; Wen et al. 2017a, b; Nigro et al. 2016; Abbasi et al. 2018; Mishra et al. 2020). However, little is known about whether PD disrupts the topological organization of grey matter (GM) morphological networks.

Structural MRI can be used to assess GM networks by calculating interregional morphological correlations to form a structural covariance network (Bassett et al. 2008; He et al. 2007). Several studies have characterized the brain structural covariance networks in PD (Xu et al. 2017, 2018; Wu et al. 2018; Chang et al. 2017; Oosterwijk et al. 2018; Chou et al. 2015; Li et al. 2017). Most have used a seed-based analysis to characterize specific networks (Chang et al. 2017; Oosterwijk et al. 2018; Chou et al. 2015; Li et al. 2017), e.g. striatal and limbic networks. Moreover, in these analyses, structural networks were calculated by constructing a single brain network for each group (Alexander-Bloch et al. 2013), thus individual networks for each subject could not be examined and correlated with clinical variables. Several methods for constructing individual morphological networks have been proposed, but have had limitations: for example a cube-based method (Tijms et al. 2012) did not allow normalization of GM networks, so that the individual subjects’ networks may differ in size, which would influence network measures (van Wijk et al. 2010). A relatively new tool, the Kullback–Leibler divergence-based similarity (KLS) method, has been developed to normalize GM networks, constructing them of the same size across subjects (Kong et al. 2014, 2015; Wang et al. 2016), which has advantages for comparative analyses such as in patient-control comparisons. Individual metrics could be examined in relation to other patient features such as α-synuclein deposits (Khairnar et al. 2017) and motor function. Studies of individual morphological networks, as well enhancing understanding of the causes and clinical significance of whole-brain GM alterations in PD, may provide a non-invasive early-stage in vivo biomarker to guide treatment planning and predict disabilities and long-term outcome (Knossalla et al. 2018).

To this end, we used KLS to investigate the topological organization of single-subject GM networks in early-stage PD. We recruited 54 patients with early-stage PD and 54 healthy controls (HC). For each subject, a morphological GM network was constructed and its topological organization characterized by a graph-based analysis. Given previous group-based evidence of more regular organized cortical networks in PD (Wu et al. 2018; Xu et al. 2017), we hypothesized that single-subject GM networks in early-stage PD would show similar disruptions compared with HC, namely higher segregation or lower integration of the morphological network. Secondly, as it is alterations in the sensorimotor network which have been most consistently reported in early-stage PD (Tessitore et al. 2014; Luo et al. 2015; Fang et al. 2017), we hypothesized that early-stage PD patients would show disturbed nodal centralities in sensorimotor regions. Third, network alterations would be related to levels of motor disability and other clinical features. Moreover, as recent studies have suggested that connectome measures have diagnostic value in brain disorders (Lei et al. 2019), we hypothesized that GM network matrices and graph-based metrics would discriminate PD patients from HC and different motor subtypes at the individual level with significant accuracy.

## Materials and methods

### Participants

This study was approved by the local ethics committee; all participants gave written informed consent. Study patients were consecutively recruited at the Movement Disorders Outpatient Clinic of West China Hospital of Sichuan University from September 2013 to January 2017. All patients met UK PD Society Brain Bank Clinical Diagnostic Criteria (Hughes et al. 1992). Clinical assessments were performed by experienced neurologists blinded to MRI results. Motor disability was assessed by the Unified PD Rating Scale (UPDRS) III (Goetz et al. 2008) and disease severity by Hoehn and Yahr stage (Hoehn and Yahr 1967); diagnostic guidelines in China define those with Hoehn and Yahr stage ≤ 2.5 as early-stage PD patients (Chen et al. 2016). Cognition was assessed using the Mini-Mental State Examination (MMSE) (Folstein et al. 1983). Exclusion criteria at recruitment included atypical Parkinsonian disorder, prior learning disability, history of other neurologic conditions including moderate or severe head injury, stroke or vascular dementia, major psychiatric or medical illness, disease duration > 3 years, H-Y stage ≥ 3, and MMSE score ˂ 24. Further details of exclusion criteria and evaluation are described in our previous paper (Suo et al. 2017). Finally, this study included 54 right-handed early-stage PD patients (19 men, 35 women). All patients were either drug-naïve (*n* = 38) or scanned in an ‘off’ state (*n* = 16) defined as ≥ 12 h after the last dose of dopaminergic medication. The antiparkinsonian drugs taken were differing combinations of levodopa, dopamine agonists and catechol-O-methyl transferase inhibitors. Antiparkinsonian medication was withdrawn for the study and all assessments (clinical, neuropsychological and neuroimaging) were done while patients were in the off state. Levodopa equivalent daily dose (LEDD) was calculated as described elsewhere (Tomlinson et al. 2010).

We recruited 54 right-handed age- and gender-matched HC participants (18 men, 36 women) from the local area by poster advertisements. They were excluded if they had any neurologic illness, as assessed by clinical evaluation and medical records, or clinically evident structural brain defects on T1- or T2-weighted images.

### Data acquisition

High-resolution T1-weighted images covering the whole brain were acquired on a 3 T MR system (Siemens Medical System, Erlangen, Germany) using a sagittal 3-dimensional magnetization-prepared rapid gradient echo sequence. Foam padding was used to minimize head motion. The scanning parameters were: repetition time 1900 ms; echo time 2.3 ms; inversion time 900 ms; slice thickness 1 mm; no inter-slice gap; 176 slices; matrix size 256 × 256; field of view 24 × 24 cm^2^; flip angle 9°. Conventional MRI protocols were performed with a fast spin-echo sequence for the structural assessment: axial T2-weighted and fluid-attenuated inversion recovery images were obtained. Sequence parameters of conventional MRI protocols are described in supplementary materials. Two neuro-radiologists verified image quality and evaluated for clinical abnormalities.

### Data preprocessing

The MRI data were preprocessed, as previously described (Kong et al. 2015), using the automated quantitative morphological analysis technique of voxel-based morphometry (VBM) (Ashburner and Friston 2000) implemented in Statistical Parametric Mapping version 12 (http://www.fil.ion.ucl.ac.uk/spm/software/spm12/). The raw MRI data were checked manually for any apparent artifacts. The individual structural data were segmented to obtain the GM images, which were normalized to MNI 152 space using the Diffeomorphic Anatomical Registration Through Exponential Lie Algebra (DARTEL) approach (Ashburner 2007), nonlinearly modulated to compensate for spatial normalization effects, then smoothed individually with a 6 mm full-width at half-maximum Gaussian kernel. To preserve the tissue volume after warping, voxel values in individual GM images were modulated by multiplying by the Jacobian determinants derived from the normalization. Finally, a GM volume map was obtained for each participant (1.5 mm isotropic voxels).

### Construction of GM brain networks

After preprocessing, the whole brain GM was parcellated into 90 regions of interest (ROIs) as nodes using the Automated Anatomical Labeling (AAL) atlas (Tzourio-Mazoyer et al. 2002). To construct the morphological GM networks we used the KLS method to define interregional morphological relations as edges/connection (Kong et al. 2014). For every subject, for each ROI, GM volume values of all the voxels were extracted, their probability density function was estimated using kernel density estimation (Wang et al. 2016), then used to calculate the probability distribution function (PDF). The Kullback–Leibler (KL) divergence was then calculated between two PDFs of each pair of ROIs. This measures the difference between two probability distributions and equates to the information lost when a probability distribution is used to approximate another (Burnham and AnderSon 2002). The standard KL divergence from distribution Q to P is calculated using:

$$D_{\text{K}\text{L}} \left( {P\|Q} \right) = \mathop \sum \limits_{i = 1}^{n} P\left( i \right)\log \frac{P\left( i \right)}{{Q\left( i \right)}}$$

However, $D_{\text{K}\text{L}} \left( {P\|Q} \right)$ is not equal to ${ }D_{\text{K}\text{L}} \left( {Q\|P} \right)$. We used instead a symmetric measure (Kong et al. 2014, 2015; Wang et al. 2016), a variant of the KL divergence calculated as:

$$D_{\text{K}\text{L}} \left( {P,Q} \right) = \mathop \sum \limits_{i = 1}^{n} \left( {P\left( i \right)\log \frac{P\left( i \right)}{{Q\left( i \right)}} + Q\left( i \right)\log \frac{Q\left( i \right)}{{P\left( i \right)}}} \right)$$

Finally, the KLS was computed as:

$${\text{K}\text{L}\text{S}}\left( {{{P}},{{Q}}} \right) = e^{{ - {\text{D}}_{\text{K}\text{L}} \left( {{{P}},{{Q}}} \right)}}$$

where *P* and *Q* are two PDFs and n is the number of sample points (*n* = 2^7^ here, as in (Wang et al. 2016)). KLS values between all possible pairs of brain regions range from 0 to 1, where 1 is for two identical distributions. Finally, the KLS-based 90 × 90 weighted undirected matrix was generated for every subject.

### Analysis of GM brain networks

We used the GRETNA (http://www.nitrc.org/projects/gretna/) toolbox to quantify the topological properties of the weighted networks. The global metrics included small-world parameters (characteristic path length *L*_p_, clustering coefficient * C*_p_, normalized characteristic path length *λ*, normalized clustering coefficient *γ*, and small-worldness σ), and network efficiency parameters (global efficiency *E*_glob_ and local efficiency *E*_loc_). The nodal metrics included nodal betweenness, nodal degree and nodal efficiency (Rubinov and Sporns 2010). To assess small-world properties, the *C*_p_ and * L*_p_ of the network were compared with those (*C*_prandom_ and *L*_prandom_) of random networks (*n* = 100) that preserve the same number of nodes, edges, and degree distribution as the real network (Wang et al. 2015). A real network would be considered small-world if it meets the following criteria: *γ* = *C*_p_/*C*_prandom_ >  > 1 and *λ* = *L*_p_/*L*_prandom_ ≈ 1.

Consistent with previous studies (Zhang et al. 2011), we selected a range of sparsity S thresholds for the GM morphological network based on the following criteria: 1) the averaged degree over all nodes of each thresholded network was larger than 2 × log (90); and 2) the small-worldness σ of the thresholded networks was larger than 1.1 for all participants. Based on these criteria, we defined S ranging from 0.1 to 0.34. For each network metric, we calculated the area under the curve (AUC) over a range of sparsity (0.10–0.34) with an interval step of 0.01, which provides a summarized scalar for topological characterization of brain networks to limit potential bias of any single threshold.

### Statistical analysis

Differences in demographic data between PD patients and HC were analyzed with the Mann–Whitney *U* test (age and education years) and *χ*^2^ test (gender). Between-group differences in the AUC of network metrics were compared using nonparametric permutation tests (5000 permutations) (Zhang et al. 2011). For nodal metric analysis, a False Discovery Rate (FDR) correction for multiple comparisons was performed to maintain a significant level of 0.05. Partial correlations taking age, gender and education years as covariates were computed to evaluate relationships between the network metrics and clinical variables (e.g. UPDRS-III, Hoehn and Yahr stage and MMSE scores) in PD.

The network-based statistics (NBS) (http://www.nitrc.org/projects/nbs/) approach was used to localize altered morphologic connection in the GM network (Zalesky et al. 2010). First, a threshold (*P* < 0.05) was applied to identify suprathreshold connections, among which any connected components and their size (the number of connections) were determined. Second, a nonparametric permutation approach was used to derive the empirical null distribution of connected component size for estimating the significance of each connected component (5000 permutations). Finally, for a connected component of size N found in the real grouping of HC and patients, its corrected p value was determined by finding the proportion of the 5000 permutations for which the maximal connected component was larger than N.

Functional brain network studies have found that different PD motor subtypes have distinct small-world characteristics (Zhang et al. 2014; Ma et al. 2017a). We, therefore, explored whether PD motor subtypes also showed morphological network differences.

### Support vector machine (SVM) analysis

To determine whether morphological network measures can detect early-stage PD at the individual level, SVM analysis was applied to the GM morphological network matrices and graph-theoretical metrics to classify PD patients vs. HC and tremor-dominant vs. akinetic–rigid subtype. The SVM model maps the input vectors to a feature space using a set of mathematical functions known as kernels (Cortes and Vapnik 1995). In this feature space, the model finds the optimum separation surface that maximizes the margin of separation between different classes within a training dataset. Once the separation surface is determined, it can be used to predict the class of new observations using an independent testing dataset. Here a linear kernel was preferred to a nonlinear one to minimize the risk of overfitting. The model was based on LIBSVM and implemented using the Scikit-Learn library (Pedregosa et al. 2012). A five-fold stratified cross-validation was used, dividing the original sample into 5 nonoverlapping folds that preserved the relative proportion of the two classes. Four folds were defined as a training set and the remaining fold as a test set in each iteration. The linear SVM has only one parameter (soft margin parameter C) that controls the trade-off between reducing training errors and having a larger separation margin. An internal cross-validation was performed to select the optimal parameter. This parameter was optimized by performing a grid search (i.e., *C* = 10^–3^, 10^–2^, 10^–1^, 10^0^, 10^1^, 10^2^, 10^3^, 10^4^) to estimate the best value. An SVM model with the optimal parameter was trained on the training set. Its performance was assessed on the test set in terms of balanced accuracy, specificity, and sensitivity. The reported balanced accuracy, specificity, and sensitivity are the mean values calculated on each partition of the cross-validation scheme. To estimate the significance for each SVM, a nonparametric permutation test were performed to calculate a P value for balanced accuracy (Golland and Fischl 2003). This involved repeating the classification procedure 1000 times with different random permutations of the group labels. A P value was then calculated by dividing the number of times that the balanced accuracy was higher for the permuted labels than the real labels by 1000.

## Results

### Demographic and clinical characteristics

There were no significant differences in age, gender, or years of education between PD patients and HC (*P* > 0.05) (Table 1).

**Table 1:** Demographics and clinical characteristics of patients with early-stage Parkinson's disease and healthy controls

	HC (*n* = 54)	PD (*n* = 54)	*P*
Age (years)	55.9 ± 6.7	56.0 ± 7.2	0.658^a^
Gender (female/male)	36/18	35/19	0.845^b^
Education (years)	9.3 ± 3.3	9.0 ± 3.6	0.776^a^
Disease duration (years)	NA	1.5 ± 0.9	NA
UPDRS III score	NA	17.4 ± 9.0	NA
MMSE score	NA	27.9 ± 1.9	NA
Hoehn and Yahr stage	NA	1.6 ± 0.5	NA
Age at onset (years)	NA	54.5 ± 7.3	NA
Side of onset (left/right/symmetric)	NA	24/28/2	NA
Motor phenotype (T/A/M)	NA	25/17/12	NA
Levodopa equivalent daily dose	NA	129.4 ± 243.1	NA

Measurements presented as mean ± SD or counts

*PD* Parkinson's disease, *HC* healthy controls, *y* years, *T* tremor-dominant, *A* akinetic–rigid, *M* mixed, *UPDRS* Unified Parkinson’s Disease Rating Scale, *MMSE* Mini–Mental State Examination, *NA* not applicable

^a^*P* values for comparisons between PD and HC using Mann–Whitney *U* test

^b^*P* value for comparison using *χ*^2^ test

### Alterations of global and nodal brain network metrics in PD

**Fig. 1:**
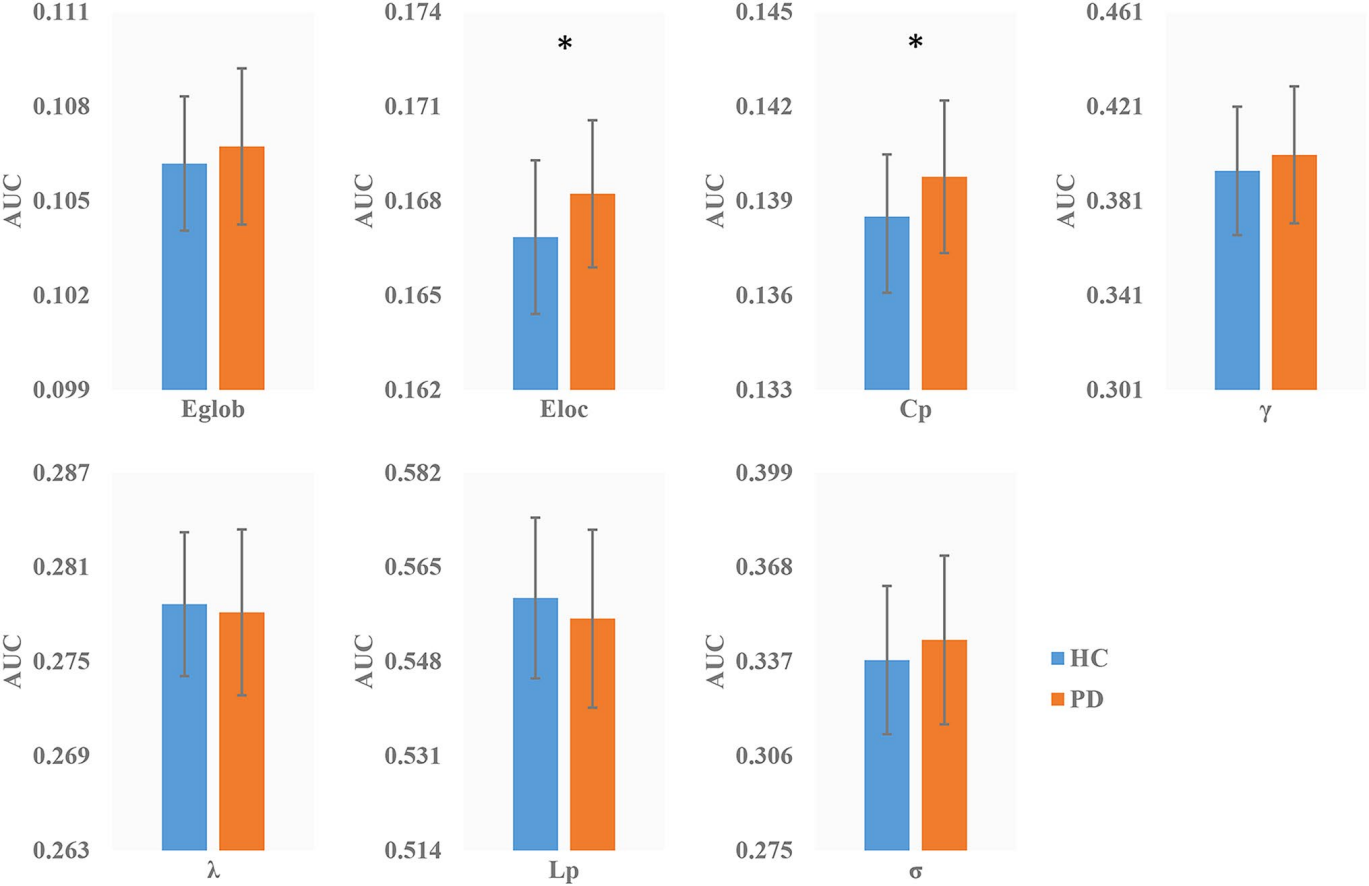
Differences in global topological properties of the brain grey matter network between PD and HC. *C*_p_ and *E*_loc_ were significantly different between the two groups. An asterisk designates network metrics with a significant difference (*P* < 0.05). *PD* Parkinson’s disease, *HC* healthy control, *C*_p_ clustering coefficient, *L*_p_ characteristic path length, *E*_glob_ global efficiency, *E*_loc_ local efficiency, *γ* normalized clustering coefficient, *λ* normalized characteristic path length, *σ* small-worldness

Both the PD and HC group showed small-world topology in the GM network (Fig S1 in supplementary materials). Compared with HC, PD showed a significantly higher * C*_p_ (*P* = 0.014) and *E*_loc_ (*P* = 0.014), with no significant differences in *L*_p_ (*P* = 0.100), *γ* (*P* = 0.104), *λ* (*P* = 0.298), *σ* (*P* = 0.098) or *E*_glob_ (*P* = 0.115) (Fig. 1, Table S1 in supplementary materials).

**Fig. 2:**
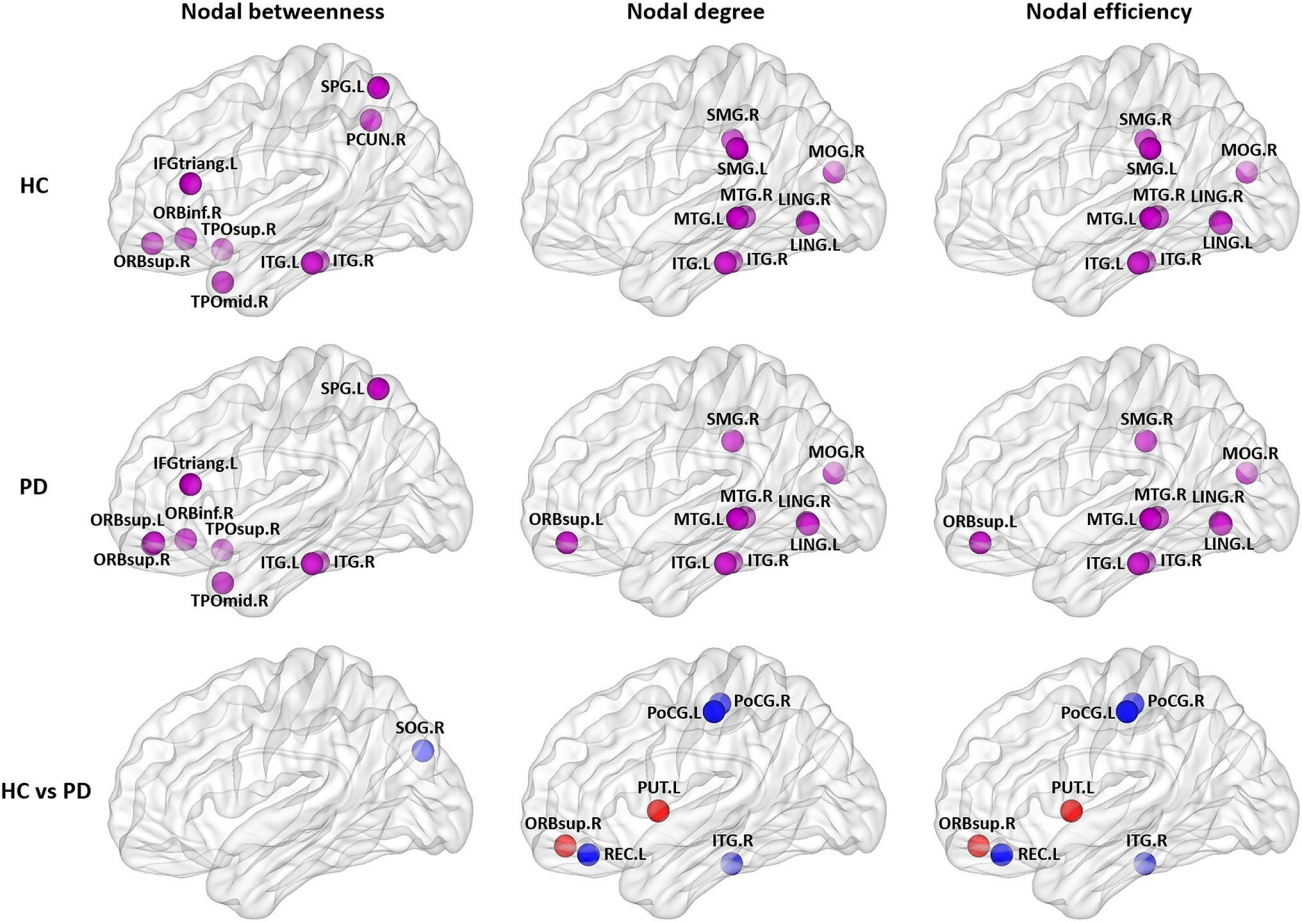
Hub regions in each group and nodal centrality differences between PD and HC. Brain regions with the highest nodal centralities (top 10%) were defined as hubs in each group and were presented in purple. Significantly lower nodal centralities in PD compared with HC were presented in blue and higher nodal centralities in red. The nodes were mapped onto the cortical surfaces using the BrainNet Viewer package (http://www.nitrc.org/projects/bnv). *L* left, *R* right, *IFGtriang* inferior frontal gyrus, triangular part, *ITG* inferior temporal gyrus, *LING* lingual gyrus, *MOG* middle occipital gyrus, *MTG* middle temporal gyrus, *ORBinf* inferior frontal gyrus, orbital part, *ORBsup* superior frontal gyrus, orbital part, *PCUN* precuneus, *PoCG* postcentral gyrus, *PUT* putamen, *REC*, gyrus rectus, *SMG* supramarginal gyrus, *SOG* superior occipital gyrus, *SPG* superior parietal gyrus, *TPOmid* temporal pole: middle temporal gyrus, *TPOsup* temporal pole: superior temporal gyrus

Fig S2 in supplementary materials show the nodal betweenness, nodal degree and nodal efficiency for all 90 cortical regions in HC and PD group. To locate brain regions that were consistently identified as hubs, we assigned each node a hub-score (ranged from 0 to 3) indicating the frequency with which the node fell within the top 10% nodes across nodal centrality metrics. In the HC group, 9 association cortex regions were identified as hub regions; in PD group, 8 association cortex and 1 paralimbic cortex regions were identified as hub regions (Fig. 2, Table S2 in supplementary materials). In both groups, 8 brain regions (bilateral middle and inferior temporal gyrus, bilateral lingual gyrus, right supramarginal gyrus and right middle occipital gyrus) were identified as hubs in common. These observations are comparable to the putative hubs reported in previous studies of morphological brain networks (Wang et al. 2016; He et al. 2007).

We identified the brain regions showing significant between-group differences in at least one nodal metric (*P* < 0.05, FDR corrected) (Table 2; Fig. 2). Compared with HC, PD showed higher nodal centralities in the right superior frontal gyrus (orbital part) and left putamen; PD showed lower nodal centralities in the left rectus gyrus, bilateral postcentral gyrus, right superior occipital gyrus, and right inferior temporal gyrus.

**Table 2:** Regions showing altered nodal centralities in patients with early-stage Parkinson’s disease compared with healthy controls

Brain regions	*P* value (Cohen’s * d*)		
	Nodal betweenness	Nodal degree	Nodal efficiency
Parkinson’s disease < Healthy controls			
Left gyrus rectus	0.0164 (− 0.384)	**0.0002** (− 0.787)	**0.0002** (− 0.787)
Right superior occipital gyrus	**0.0026** (− 0.556)	0.0990 (− 0.247)	0.1320 (− 0.214)
Left postcentral gyrus	0.1250 (0.229)	**0.0026** (− 0.558)	**0.0012** (− 0.568)
Right postcentral gyrus	0.1348 (0.220)	**0.0024** (− 0.573)	**0.0022** (− 0.589)
Right inferior temporal gyrus	0.1856 (− 0.177)	**0.0024** (− 0.561)	**0.0016** (− 0.575)
Parkinson’s disease > Healthy controls			
Right superior frontal gyrus, orbital part	0.0780 (0.271)	**0.0004** (0.675)	**0.0004** (0.653)
Left putamen	0.0056 (0.477)	**0.0024** (0.591)	**0.0004** (0.625)

Regions were considered abnormal in patients if they exhibited significant between-group differences (*P* < 0.05, FDR corrected) in at least one of the three nodal centralities (shown in bold font)

### Alterations in network connection in PD

**Fig. 3:**
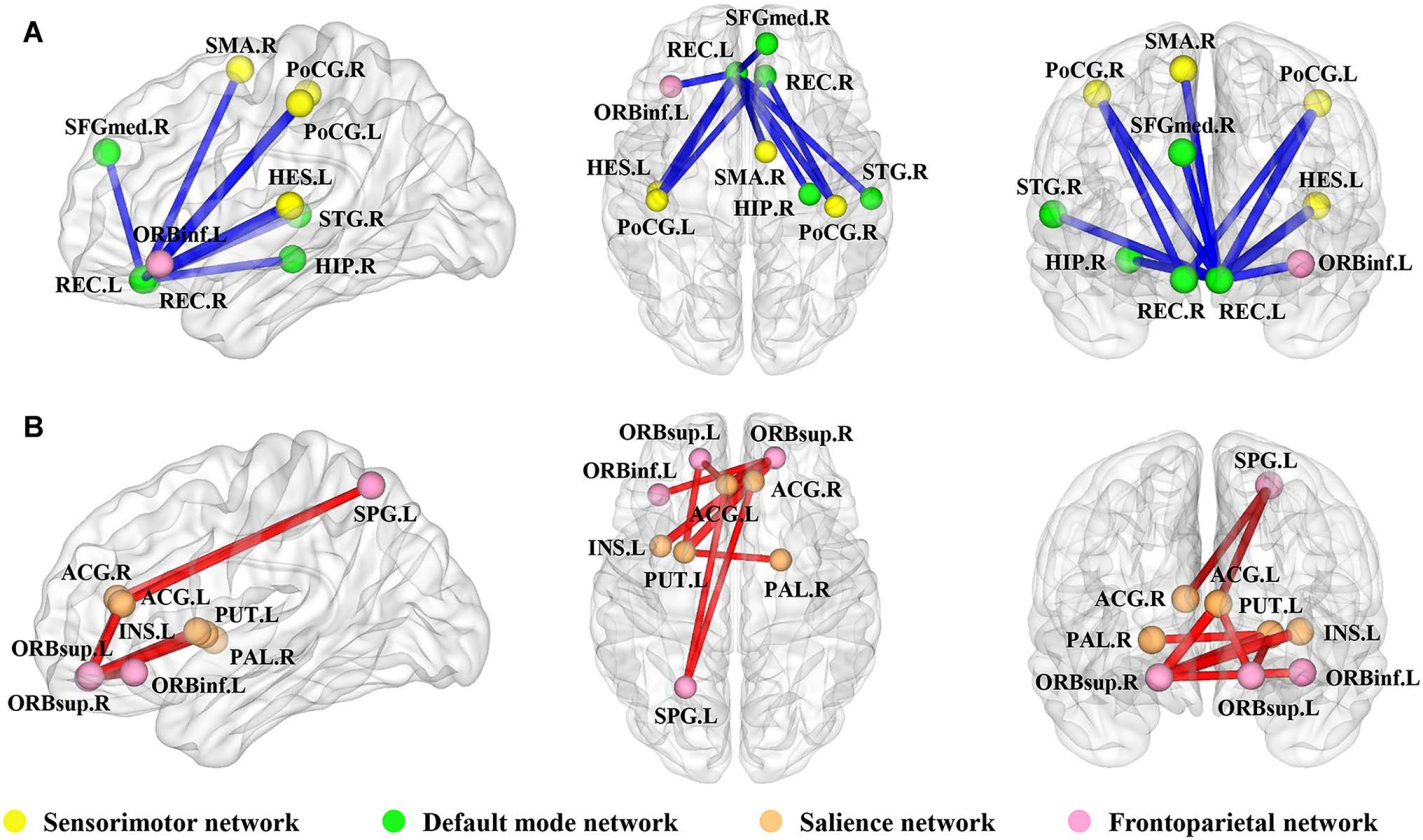
PD-related alterations in the network connection. Each node denotes a brain region and each line a connection. Significantly decreased connections in PD compared with HC is presented in blue and increased connections in red. Associations of these nodes with specific brain networks are shown in different color, sensorimotor network (in yellow), default mode network (in green), salience network (in orange), and frontoparietal network (in pink). *L* left, *R* right, *ORBsup* superior frontal gyrus, orbital part, *ORBinf* inferior frontal gyrus, orbital part, *SFGmed* superior frontal gyrus, medial, *SMA* supplementary motor area, *REC* rectus gyrus, *INS* insula, *ACG* anterior cingulate gyrus, *HIP* hippocampus, *PoCG* postcentral gyrus, *SPG* superior parietal gyrus, *PUT* putamen, *PAL* palladium, *HES* Heschl gyrus, *STG* superior temporal gyrus

Compared with HC, PD presented both decreased and increased morphological connections in the NBS analysis (*P* < 0.05, NBS corrected). The subnetwork with significantly decreased connections consisted of 10 brain regions and 10 edges, mainly involved in the default mode network (DMN) and the sensorimotor network (Fig. 3a). The subnetwork with significantly increased connections contained 9 brain regions and 9 edges, mainly involved in the frontoparietal network and the salience network (Fig. 3b).

### Relationships between network metrics and clinical variables in PD

UPDRS-III values were negatively correlated with the nodal efficiency of right postcentral gyrus (*P* = 0.021, *r* = − 0.321) and the nodal degree of right postcentral gyrus (*P* = 0.033, *r* = − 0.299); Hoehn and Yahr stage was negatively correlated with the nodal efficiency of right postcentral gyrus (*P* = 0.015, *r* = − 0.340), nodal degree of right postcentral gyrus (*P* = 0.031, *r* = − 0.302), and nodal betweenness of right superior occipital gyrus (*P* = 0.009, *r* = − 0.363) (Fig S3). However, these correlations did not survive multiple comparison corrections. No significant association was identified with other network metrics or between MMSE score and any network metrics. Excluding two patients with a symmetric side of onset, the main findings of the correlation analyses were maintained (Fig S4).

Multiple linear regression analysis revealed no significant associations between UPDRS-III scores and Hoehn and Yahr stage after correction for multiple comparisons at FDR < 0.05 (Table S3).

### Single-subject classification of PD patients and HC

Using graph-based metrics, the mean balanced accuracy of classification of PD patients versus HC was 72.7%, with sensitivity 63.6% and specificity 81.8% (*P* = 0.026). Using GM network matrices, discrimination of PD patients from HC at the single-subject level was improved; the mean balanced accuracy was 73.1%, with sensitivity 72.4% and specificity 73.8% (*P* < 0.001).

**Fig. 4:**
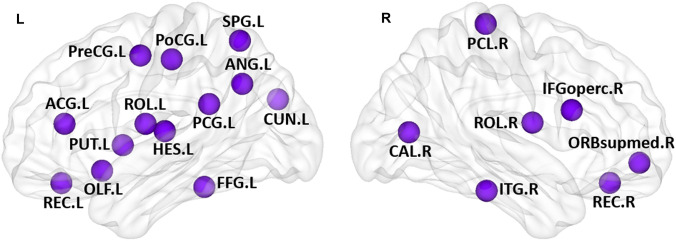
Twenty brain regions making the greatest contribution to the single-subject classification of PD vs. HC. The nodes were mapped onto the cortical surfaces using the BrainNet Viewer package (http://www.nitrc.org/projects/bnv). *PD* Parkinson’s disease, *HC* healthy control, *L* left, *R* right, *ACG* anterior cingulate and paracingulate gyri, *ANG* angular gyrus, *CAL* calcarine fissure and surrounding cortex, *CUN* cuneus, *FFG* fusiform gyrus, *HES* Heschl gyrus, *IFGoperc* inferior frontal gyrus, opercular part, *ITG* inferior temporal gyrus, *OLF* olfactory cortex, *ORBsupmed* superior frontal gyrus, medial orbital, *PCG* posterior cingulate gyrus, *PCL* paracentral lobule, *PoCG* postcentral gyrus, *PreCG* precentral gyrus, *PUT* putamen, *REC* gyrus rectus, *ROL* Rolandic operculum, *SPG* superior parietal gyrus

To identify brain regions providing the greatest contribution to single-subject classification, the mean absolute value of the weights of the model across the different folds of the cross-validation were calculated. The 20 brain regions with the highest mean values are reported in Table 3 and shown in Fig. 4. It can be seen that most of the brain regions overlap with the brain regions showing significant between-group differences in nodal metrics.

**Table 3:** Top 20 most relevant brain regions for the classification analysis of patients and controls

No	Regions	Abbreviations
1	Right paracentral lobule	PCL.R
2	Left Heschl gyrus	HES.L
3	Left gyrus rectus	REC.L
4	Left anterior cingulate and paracingulate gyri	ACG.L
5	Right rolandic operculum	ROL.R
6	Right superior frontal gyrus, medial orbital	ORBsupmed.R
7	Left fusiform gyrus	FFG.L
8	Left olfactory cortex	OLF.L
9	Right calcarine fissure and surrounding cortex	CAL.R
10	Right inferior temporal gyrus	ITG.R
11	Right inferior frontal gyrus, opercular part	IFGoperc.R
12	Right posterior cingulate gyrus	PCG.R
13	Left postcentral gyrus	PoCG.L
14	Left angular gyrus	ANG.L
15	Right gyrus rectus	REC.R
16	Left rolandic operculum	ROL.L
17	Left precentral gyrus	PreCG.L
18	Left lenticular nucleus, putamen	PUT.L
19	Left superior parietal gyrus	SPG.L
20	Left cuneus	CUN.L

All the brain regions are from automated anatomical labelling atlas (Tzourio-Mazoyer et al. 2002)

### Brain network measures between different PD motor subtypes

There were no significant differences in the global, nodal or connection characteristics between tremor-dominant and akinetic–rigid motor subtypes (*P* > 0.05). As shown in Table S4, the mean balanced accuracy of the two motor subtypes’ classification using GM network matrices was close to the chance level (*P* > 0.05), but using graph-based metrics, the mean balanced accuracy of classification of two motor subtypes was 67.0%, with sensitivity 50.0% and specificity 84.0% (*P* = 0.031). The 20 brain regions with the highest mean values are reported in Table S5.

## Discussion

This study demonstrated significant alterations, compared to HC, in the topological properties of single-subject whole-brain GM networks in early-stage PD. They showed higher network segregation, reflected by higher *C*_p_ and *E*_loc_, and altered nodal centralities in the putamen and temporal-occipital regions. Analysis of individual patient networks revealed that nodal centralities in right postcentral gyrus were negatively correlated with UPDRS-III scores and Hoehn and Yahr stage. PD showed altered subnetworks, with decreased morphological connections in DMN and sensorimotor network and increased connections mainly in frontoparietal and salience networks. Lastly, GM network matrices and graph-based metrics show the potential for allowing single-subject classification of PD patients and HC with significant accuracy of 73.1 and 72.7%, while graph-based metrics allowed single-subject classification of tremor-dominant and akinetic–rigid motor subtypes with significant accuracy of 67.0%. Our findings might provide new insights into the understanding of the pathophysiology of PD.

The neurobiological meaning of morphological similarity networks is not completely understood. One possible mechanism driving this level of brain network organization is axonal tension theory, which proposes that similarity of regions results during morphogenesis from mechanical tension in the axons between them (Van Essen 1997). Note that these GM networks cannot be simply taken as a proxy measure for fiber connections; morphological similarity may contain specific information (e.g. cytoarchitectonic similarity and co-expression of specialized neuronal function genes) unrelated to connectivity (Seidlitz et al. 2018), and other factors such as levels of neurophysiological activity can drive morphological similarity, regardless of direct white matter connections. Healthy human brain cortical morphology is a complex but efficient network which balances local specialization and global integration to maximize parallel information processing (Wang et al. 2016). However, pathological lesions in PD, e.g. misfolded proteins which deposit in cortical GM and induce GM density changes (Khairnar et al. 2017; McMillan and Wolk 2016), might impact the morphological similarity of different GM regions, and thus disturb the distributed GM morphological network architecture.

In formal terms, the brain’s small-world organization strikes an optimal balance between network segregation (reflected by *C*_p_, *γ* and *E*_loc_) and integration (reflected by *L*_p_, *λ* and *E*_glob_) of information processing. Despite an overall small-world architecture similar to HC, PD patients showed higher *C*_p_ and *E*_loc_, which represent higher segregation of GM networks. The network integration measures (*L*_p_, *λ* and *E*_glob_) of early-stage PD patients were preserved, in contrast to previous findings of longer *L*_p_ and lower E_glob_ in advanced PD (Gottlich et al. 2013; Suo et al. 2017). This may mean that PD patients are initially able to maintain overall information transfer, but that as the disease progress, brain networks gradually fail to maintain global integration. From the perspectives of segregation and integration, altered small-world properties in disease can be thought of falling into four patterns: regularization, randomization, and stronger and weaker small-worldization (Suo et al. 2018). Taken together, the higher segregation and preserved integration of GM networks may indicate a regularization pattern of altered small-world properties in early-stage PD, which is consistent with the previous findings in structural co-variance networks (Wu et al. 2018; Xu et al. 2017) and individual morphological network studies (Zhang et al. 2015a).

At a regional level, higher nodal centralities were observed in the left putamen. In PD, the prominent degeneration of dopaminergic nigrostriatal neurons of the substantia nigra leads to dopamine deficiency in the striatum, especially in the putamen, which is functionally interposed between the cortex and thalamus to modulate movement execution. In addition, lower nodal centralities in PD were observed in the postcentral gyrus, which were negatively correlated with the UPDRS-III scores, similar to our previous functional network study finding with a substantially overlapping sample (Suo et al. 2017). In line with previous work (Sang et al. 2015; Suo et al. 2017; Luo et al. 2015; Xu et al. 2018), our results indicate that the putamen and postcentral gyrus, which are associated with sensorimotor dysfunction of PD, may be disrupted at an early stage. Further, our data highlight that at a morphological network level, nodal connections of these regions to other brain areas may be altered early in the course of disease progression.

Lower nodal centralities were also found in the superior occipital gyrus and inferior temporal gyrus, which comprise the visuoperceptive pathway responsible for the representation of complex object features and facial perception. Abnormalities of grey matter (Goldman et al. 2014), neuronal activity (Meppelink et al. 2009), and nodal centralities (Luo et al. 2015) in temporal-occipital regions have been reported in PD patients compared with HC, even before visual symptoms become clinically evident. Lower metabolism of the visual cortex was found in a different motor subtype of PD, suggesting that PD patients may control their motor symptoms through visual information (Hu et al. 2019). Our results suggest that impaired visual processing might occur early, before the recognized visuospatial dysfunction in early-stage PD patients.

Our examination of morphological connections of pairs of brain regions using NBS tools revealed a subnetwork with decreased morphological connections affecting the DMN and the sensorimotor network. It is unsurprising that there are abnormal connections associated with sensorimotor network in PD. As for the DMN, which plays a crucial role in emotion regulation and cognitive process (Menon 2011), previous studies have found disrupted connections not only in PD patients with cognitive deficits (Pereira et al. 2015), but also in cognitively unimpaired PD patients (Tessitore et al. 2012). Recent research on cognitive impairment in PD reveals that DMN disruption characterizes PD patients, regardless of cognitive status, suggesting that DMN may be altered in PD as part of the pathological changes associated with PD independently of the development of clinically evident mild cognitive decline (Amboni et al. 2015). Notably, our patients were cognitively intact as judged by the MMSE score. These data, taken together with previous findings (Tessitore et al. 2012; Sandrone and Catani 2013; Suo et al. 2017), suggest that DMN and sensorimotor network disruption might be a common feature of PD, perhaps a direct consequence of the pathological brain damage in PD. Neurochemically, PD is characterized by depletion of an important neurotransmitter, which can be reasonably assumed to reflect reduced connectivity of specific connections. In contrast, increased morphological connections were observed in PD mainly involving the frontoparietal and salience network, which subserve cognitive and executive functions (Menon 2011). Our findings are consistent with numerous studies showing greater activation (Caminiti et al. 2015; Marie et al. 2007), and increased centrality (Zhang et al. 2015b) and functional connectivity (de Schipper et al. 2018; Navalpotro-Gomez et al. 2020) in PD. These increases may perhaps reflect cerebral compensation for primary pathophysiological changes. Given our patients’ relatively intact neuropsychological abilities, we speculate that increased morphological connections in these two networks might be an early compensatory mechanism for disease-related deficits, perhaps to maintain cognitive performance. This is of course speculative, and more studies are required on this issue.

Overall, these global, nodal and connection findings support the conceptualization of PD as a network disease, an impairment in the normal balance of brain networks. *C*_p_ is calculated as $C_{{\text{p}}} = \frac{1}{n}\mathop \sum \nolimits_{i \in N} \frac{{2t_{{\text{i}}} }}{{k_{{\text{i}}} \left( {k_{{\text{i}}} - 1} \right)}}$, *n* is the number of nodes (*n* = 90 in our study), *t*_i_ is the number of triangles around a node *i*, and *k*_i_ is the number of links connected to a node * i* known as nodal degree (Rubinov and Sporns 2010). The mean *C*_p_ for the network hence reflects, on average, the prevalence of clustered connectivity around individual nodes, which is normalized individually for each node. From the above formula (Rubinov and Sporns 2010), it can be seen that a decrease of node centrality could lead to the increase of *C*_p_, which has been also observed in prior studies (Wu et al. 2018; Nan et al. 2016; Suo et al. 2015; Zhang et al. 2012). Higher *C*_p_ and *E*_loc_, representing a higher local segregation of the networks, suggest a shift toward a topological pattern of regularization. Although the biological mechanisms of the shift remain unclear, the regular pattern has been shown to reduce signal propagation speed and synchronizability across distant regions compared with small-world networks (Strogatz 2001). On examination of the PD-related network, a considerable proportion of the increased connections are short-range (i.e. cortical-subcortical connections), whereas decreased connections were mainly long-range (i.e. different cortex connections, especially between the frontal and temporal cortex, and the frontal and parietal cortex). The abnormalities in the short-range connections might be the predominant contributors to the segregation alterations of topological organization (He et al. 2009), which have been observed in other neurodegenerative diseases such as amyotrophic lateral sclerosis (Zhang et al. 2019). The network connections identified in PD patients by NBS comprised mainly regions that overlap spatially the regions with altered nodal centralities, which are directly related to the whole-brain global network topology. Taken together, these topological alterations at different levels reflect the deficient transmission of information, and are related to disability in PD through its influence on sensorimotor coordination.

Consistent with our third hypothesis, the accuracy of classification of PD vs. HC was 73.1 and 72.7% using network matrices and graph-based metrics, respectively. Our recent study of schizophrenia also found that connectome-wide matrices allowed single-subject classification of patients and controls with high accuracy (average: 81%) (Lei et al. 2019). Moreover, brain regions showing the between-group difference in nodal metrics we observed overlapped with most of the top 20 brain regions providing the greatest contribution to the classification: among them were the above-mentioned sensorimotor and visual regions. Therefore, the current results provide support for the view that brain networks based on structural MRI might have potential diagnostic value for PD. Moreover, although the accuracy of the classification of tremor-dominant vs. akinetic-rigid subtypes was 67.0%, this does suggest that graph-based metrics might help to differentiate different subtypes of PD patients. It is important to acknowledge that, at present, neuroimaging is still far from becoming a tool used in the day-to-day clinical practice. This is because there is still insufficient evidence that such methodology could reliably support diagnostic and prognostic evaluations. Nevertheless, the present study enhances current understanding of network-level abnormalities in PD, which in turn could inform the development of diagnostic and prognostic imaging-based markers in the future.

Our study has several limitations. First, further studies will be needed to determine whether these altered network patterns can be replicated and whether they change with disease progression and treatment. Second, there is no widely accepted optimal approach for defining nodes and edges. We used the AAL template regions as nodes and interregional similarity in the distributions of regional GM volume as edges, but other node definitions and surface-based morphological measures (such as cortical thickness or surface area) could also be used to calculate network metrics. Anatomical boundaries in the AAL template may not necessarily match functional boundaries, and nodes could be defined functionally rather than anatomically. A systematic comparison of parcellation methods found none that optimally addressed all challenges (Arslan et al. 2018), and method development research is ongoing. As most work has used the AAL template, we made that methodologically conservative choice to facilitate literature comparison. Future research should validate and extend our observations across multiple resolutions, using different templates and approaches for combining nodes into networks. Third, although the patients in this study were restricted to the early stage, their heterogeneity may have influenced the network topology. Future studies with a more homogeneous sample might refine our conclusions. Our subgroup analysis showed no significant difference between motor subtypes, and this factor is unlikely to explain the observed differences in GM morphological networks. However, non-motor symptoms such as psychiatric symptoms were not evaluated. Fourth, we used MMSE to exclude global cognitive impairment or dementia. There are more sensitive neuropsychological tests that could provide a more precise characterization of the relationship of cognitive symptoms to network changes early in PD. Future studies will delineate specific subtype-related brain network reorganization following a detailed assessment of cognitive and affective symptoms. Fifth, the results of correlation analyses between network metrics and clinical variables in PD patients did not survive multiple comparison corrections, and should be considered exploratory. To increase statistical power, future studies must be conducted using a larger sample of PD patients with strict inclusion and criteria. Finally, available connectome studies are restricted to the macroscopic scale and do not provide information on the functionally important microscopic dimension. In research using the ultrahigh-resolution brain model BigBrain (Amunts et al. 2013) to construct a brain network, the similarity of regional distribution in microscopic data may be a concise and meaningful measure of connections at an almost cellular level. Thus, it would be interesting to explore underlying patterns further by combining microscopic scale techniques from the perspective of complex networks.

In conclusion, the use of single-subject GM networks may extend previous group-level morphological brain network analyses by showing their relation to disease severity and their potential diagnostic value, and providing a potential structural basis for the functional alterations observed in our previous work with an overlapping patient sample (Suo et al. 2017). PD was characterized by sub-optimal topological organization of GM networks, reflected in higher network segregation at an early stage of illness, suggesting how GM networks might contribute to imaging evidence for the classification of individual PD patients. These results demonstrate the potential of graph theoretical measures of the brain network as imaging biomarkers for understanding and characterizing PD. Specifically, this study adds to the field of psychoradiology (Sun et al. 2018; Huang et al. 2019; Gong 2020), an evolving subspecialty of radiology, which is primed to be of major clinical importance in guiding diagnostic and therapeutic decision making in patients with neuropsychiatric disorders.

## Supplementary Information

Below is the link to the electronic supplementary material.

Supplementary file1 (DOCX 846 KB)

## References

[CR1] Aarabi MH, Kamalian A, Mohajer B, Shandiz MS, Eqlimi E, Shojaei A, Safabakhsh H (2015). A statistical approach in human brain connectome of Parkinson Disease in elderly people using Network Based Statistics. Conf Proc IEEE Eng Med Biol Soc.

[CR2] Abbasi N, Mohajer B, Abbasi S, Hasanabadi P, Abdolalizadeh A, Rajimehr R (2018). Relationship between cerebrospinal fluid biomarkers and structural brain network properties in Parkinson's disease. Mov Disord.

[CR3] Alexander-Bloch A, Giedd JN, Bullmore E (2013). Imaging structural co-variance between human brain regions. Nat Rev Neurosci.

[CR4] Amboni M, Tessitore A, Esposito F, Santangelo G, Picillo M, Vitale C, Giordano A, Erro R, de Micco R, Corbo D, Tedeschi G, Barone P (2015). Resting-state functional connectivity associated with mild cognitive impairment in Parkinson's disease. J Neurol.

[CR5] Amunts K, Lepage C, Borgeat L, Mohlberg H, Dickscheid T, Rousseau ME, Bludau S, Bazin PL, Lewis LB, Oros-Peusquens AM, Shah NJ, Lippert T, Zilles K, Evans AC (2013). BigBrain: an ultrahigh-resolution 3D human brain model. Science.

[CR6] Arslan S, Ktena SI, Makropoulos A, Robinson EC, Rueckert D, Parisot S (2018). Human brain mapping: a systematic comparison of parcellation methods for the human cerebral cortex. Neuroimage.

[CR7] Ashburner J (2007). A fast diffeomorphic image registration algorithm. Neuroimage.

[CR8] Ashburner J, Friston KJ (2000). Voxel-based morphometry–the methods. Neuroimage.

[CR9] Atkinson-Clement C, Pinto S, Eusebio A, Coulon O (2017). Diffusion tensor imaging in Parkinson's disease: Review and meta-analysis. Neuroimage Clin.

[CR10] Bassett DS, Bullmore E, Verchinski BA, Mattay VS, Weinberger DR, Meyer-Lindenberg A (2008). Hierarchical organization of human cortical networks in health and schizophrenia. J Neurosci.

[CR11] Berman BD, Smucny J, Wylie KP, Shelton E, Kronberg E, Leehey M, Tregellas JR (2016). Levodopa modulates small-world architecture of functional brain networks in Parkinson's disease. Mov Disord.

[CR12] Burnham K, AnderSon D (2002). Model selection and multimodel inference: a practical information-theoretic approach.

[CR13] Caminiti SP, Siri C, Guidi L, Antonini A, Perani D (2015). The neural correlates of spatial and object working memory in elderly and Parkinson's disease subjects. Behav Neurol.

[CR14] Chang YT, Lu CH, Wu MK, Hsu SW, Huang CW, Chang WN, Lien CY, Lee JJ, Chang CC (2017). Salience network and depressive severities in Parkinson's disease with mild cognitive impairment: a structural covariance network analysis. Front Aging Neurosci.

[CR15] Chen S, Chan P, Sun S, Chen H, Zhang B, Le W, Liu C, Peng G, Tang B, Wang L, Cheng Y, Shao M, Liu Z, Wang Z, Chen X, Wang M, Wan X, Shang H, Liu Y, Xu P, Wang J, Feng T, Chen X, Hu X, Xie A, Xiao Q (2016). The recommendations of Chinese Parkinson's disease and movement disorder society consensus on therapeutic management of Parkinson's disease. Transl Neurodegener.

[CR16] Chou KH, Lin WC, Lee PL, Tsai NW, Huang YC, Chen HL, Cheng KY, Chen PC, Wang HC, Lin TK, Li SH, Lin WM, Lu CH, Lin CP (2015). Structural covariance networks of striatum subdivision in patients with Parkinson's disease. Hum Brain Mapp.

[CR17] Cortes C, Vapnik V (1995). Support-vector networks. Mach Learn.

[CR18] Craddock RC, Jbabdi S, Yan CG, Vogelstein JT, Castellanos FX, Di Martino A, Kelly C, Heberlein K, Colcombe S, Milham MP (2013). Imaging human connectomes at the macroscale. Nat Methods.

[CR19] de Schipper LJ, Hafkemeijer A, van der Grond J, Marinus J, Henselmans JML, van Hilten JJ (2018). Altered whole-brain and network-based functional connectivity in Parkinson's disease. Front Neurol.

[CR20] Fang J, Chen H, Cao Z, Jiang Y, Ma L, Ma H, Feng T (2017). Impaired brain network architecture in newly diagnosed Parkinson's disease based on graph theoretical analysis. Neurosci Lett.

[CR21] Folstein MF, Robins LN, Helzer JE (1983). The mini-mental state examination. Arch Gen Psychiatry.

[CR22] Galantucci S, Agosta F, Stefanova E, Basaia S, van den Heuvel MP, Stojkovic T, Canu E, Stankovic I, Spica V, Copetti M, Gagliardi D, Kostic VS, Filippi M (2017). Structural brain connectome and cognitive impairment in Parkinson disease. Radiology.

[CR23] Goetz CG, Tilley BC, Shaftman SR, Stebbins GT, Fahn S, Martinez-Martin P, Poewe W, Sampaio C, Stern MB, Dodel R, Dubois B, Holloway R, Jankovic J, Kulisevsky J, Lang AE, Lees A, Leurgans S, LeWitt PA, Nyenhuis D, Olanow CW, Rascol O, Schrag A, Teresi JA, van Hilten JJ, LaPelle N, Movement Disorder Society URTF (2008). Movement Disorder Society-sponsored revision of the Unified Parkinson's Disease Rating Scale (MDS-UPDRS): scale presentation and clinimetric testing results. Mov Disord.

[CR24] Goldman JG, Stebbins GT, Dinh V, Bernard B, Merkitch D, deToledo-Morrell L, Goetz CG (2014). Visuoperceptive region atrophy independent of cognitive status in patients with Parkinson's disease with hallucinations. Brain.

[CR25] Golland P, Fischl B (2003). Permutation tests for classification: towards statistical significance in image-based studies. Inf Process Med Imaging.

[CR94] Gong Q (2020) Psychoradiology, Neuroimaging Clinics of North America, vol 30. Elsevier Inc, New York, pp 1–123. https://www.elsevier.com/books/psychoradiology-an-issue-of-neuroimaging-clinics-of-north-america/gong/978-0-323-70886-9

[CR26] Gottlich M, Munte TF, Heldmann M, Kasten M, Hagenah J, Kramer UM (2013). Altered resting state brain networks in Parkinson's disease. PLoS ONE.

[CR27] He Y, Chen ZJ, Evans AC (2007). Small-world anatomical networks in the human brain revealed by cortical thickness from MRI. Cereb Cortex.

[CR28] He Y, Dagher A, Chen Z, Charil A, Zijdenbos A, Worsley K, Evans A (2009). Impaired small-world efficiency in structural cortical networks in multiple sclerosis associated with white matter lesion load. Brain.

[CR29] Hoehn MM, Yahr MD (1967). Parkinsonism: onset, progression and mortality. Neurology.

[CR30] Hu J, Xiao C, Gong D, Qiu C, Liu W, Zhang W (2019). Regional homogeneity analysis of major Parkinson's disease subtypes based on functional magnetic resonance imaging. Neurosci Lett.

[CR93] Huang X, Gong Q, Sweeney JA, Biswal BB (2019). Progress in psychoradiology, the clinical application of psychiatric neuroimaging. Brit J Radiol.

[CR31] Hughes AJ, Daniel SE, Kilford L, Lees AJ (1992). Accuracy of clinical diagnosis of idiopathic Parkinson's disease: a clinico-pathological study of 100 cases. J Neurol Neurosurg Psychiatry.

[CR32] Jankovic J (2008). Parkinson's disease: clinical features and diagnosis. J Neurol Neurosurg Psychiatry.

[CR33] Ji GJ, Hu P, Liu TT, Li Y, Chen X, Zhu C, Tian Y, Chen X, Wang K (2018). Functional connectivity of the corticobasal ganglia-thalamocortical network in Parkinson disease: a systematic review and meta-analysis with cross-validation. Radiology.

[CR34] Kamagata K, Zalesky A, Hatano T, Di Biase MA, El Samad O, Saiki S, Shimoji K, Kumamaru KK, Kamiya K, Hori M, Hattori N, Aoki S, Pantelis C (2018). Connectome analysis with diffusion MRI in idiopathic Parkinson's disease: evaluation using multi-shell, multi-tissue, constrained spherical deconvolution. Neuroimage Clin.

[CR35] Khairnar A, Ruda-Kucerova J, Szabo N, Drazanova E, Arab A, Hutter-Paier B, Neddens J, Latta P, Starcuk Z, Rektorova I (2017). Early and progressive microstructural brain changes in mice overexpressing human alpha-Synuclein detected by diffusion kurtosis imaging. Brain Behav Immun.

[CR36] Knossalla F, Kohl Z, Winkler J, Schwab S, Schenk T, Engelhorn T, Doerfler A, Golitz P (2018). High-resolution diffusion tensor-imaging indicates asymmetric microstructural disorganization within substantia nigra in early Parkinson's disease. J Clin Neurosci.

[CR37] Kong XZ, Liu Z, Huang L, Wang X, Yang Z, Zhou G, Zhen Z, Liu J (2015). Mapping individual brain networks using statistical similarity in regional morphology from MRI. PLoS ONE.

[CR38] Kong XZ, Wang X, Huang L, Pu Y, Yang Z, Dang X, Zhen Z, Liu J (2014). Measuring individual morphological relationship of cortical regions. J Neurosci Methods.

[CR39] Lei D, Pinaya WHL, van Amelsvoort T, Marcelis M, Donohoe G, Mothersill DO, Corvin A, Gill M, Vieira S, Huang X, Lui S, Scarpazza C, Young J, Arango C, Bullmore E, Qiyong G, McGuire P, Mechelli A (2019). Detecting schizophrenia at the level of the individual: relative diagnostic value of whole-brain images, connectome-wide functional connectivity and graph-based metrics. Psychol Med.

[CR40] Li X, Xing Y, Schwarz ST, Auer DP (2017). Limbic grey matter changes in early Parkinson's disease. Hum Brain Mapp.

[CR41] Liao X, Vasilakos AV, He Y (2017). Small-world human brain networks: perspectives and challenges. Neurosci Biobehav Rev.

[CR42] Luo CY, Guo XY, Song W, Chen Q, Cao B, Yang J, Gong QY, Shang HF (2015). Functional connectome assessed using graph theory in drug-naive Parkinson's disease. J Neurol.

[CR43] Ma LY, Chen XD, He Y, Ma HZ, Feng T (2017). Disrupted brain network hubs in subtype-specific Parkinson's disease. Eur Neurol.

[CR44] Ma Q, Huang B, Wang J, Seger C, Yang W, Li C, Wang J, Feng J, Weng L, Jiang W, Huang R (2017). Altered modular organization of intrinsic brain functional networks in patients with Parkinson's disease. Brain Imaging Behav.

[CR45] Marie RM, Lozza C, Chavoix C, Defer GL, Baron JC (2007). Functional imaging of working memory in Parkinson's disease: compensations and deficits. J Neuroimaging.

[CR46] McMillan CT, Wolk DA (2016). Presence of cerebral amyloid modulates phenotype and pattern of neurodegeneration in early Parkinson's disease. J Neurol Neurosurg Psychiatry.

[CR47] Menon V (2011). Large-scale brain networks and psychopathology: a unifying triple network model. Trends Cogn Sci.

[CR48] Meppelink AM, de Jong BM, Renken R, Leenders KL, Cornelissen FW, van Laar T (2009). Impaired visual processing preceding image recognition in Parkinson's disease patients with visual hallucinations. Brain.

[CR49] Mishra VR, Sreenivasan KR, Yang Z, Zhuang X, Cordes D, Mari Z, Litvan I, Fernandez HH, Eidelberg D, Ritter A, Cummings JL, Walsh RR (2020). Unique white matter structural connectivity in early-stage drug-naive Parkinson disease. Neurology.

[CR50] Nan J, Zhang L, Zhu F, Tian X, Zheng Q, Deneen KM, Liu J, Zhang M (2016). Topological alterations of the intrinsic brain network in patients with functional dyspepsia. J Neurogastroenterol Motil.

[CR51] Navalpotro-Gomez I, Kim J, Paz-Alonso PM, Delgado-Alvarado M, Quiroga-Varela A, Jimenez-Urbieta H, Carreiras M, Strafella AP, Rodriguez-Oroz MC (2020). Disrupted salience network dynamics in Parkinson's disease patients with impulse control disorders. Parkinsonism Relat Disord.

[CR52] Nigro S, Riccelli R, Passamonti L, Arabia G, Morelli M, Nistico R, Novellino F, Salsone M, Barbagallo G, Quattrone A (2016). Characterizing structural neural networks in de novo Parkinson disease patients using diffusion tensor imaging. Hum Brain Mapp.

[CR53] Oosterwijk CS, Vriend C, Berendse HW, van der Werf YD, van den Heuvel OA (2018). Anxiety in Parkinson's disease is associated with reduced structural covariance of the striatum. J Affect Disord.

[CR54] Pan P, Zhan H, Xia M, Zhang Y, Guan D, Xu Y (2017). Aberrant regional homogeneity in Parkinson's disease: a voxel-wise meta-analysis of resting-state functional magnetic resonance imaging studies. Neurosci Biobehav Rev.

[CR55] Pedregosa F, Varoquaux G, Gramfort A, Michel V, Thirion B, Grisel O, Blondel M, Prettenhofer P, Weiss R, Dubourg V, Vanderplas J, Passos A, Cournapeau D, Brucher M, Perrot M, Duchesnay É (2012). Scikit-learn: machine learning in python. J Mach Learn Res.

[CR56] Pereira JB, Aarsland D, Ginestet CE, Lebedev AV, Wahlund LO, Simmons A, Volpe G, Westman E (2015). Aberrant cerebral network topology and mild cognitive impairment in early Parkinson's disease. Hum Brain Mapp.

[CR57] Rubinov M, Sporns O (2010). Complex network measures of brain connectivity: uses and interpretations. Neuroimage.

[CR58] Sandrone S, Catani M (2013). Journal Club. Default-mode network connectivity in cognitively unimpaired patients with Parkinson disease. Neurology.

[CR59] Sang L, Zhang J, Wang L, Zhang J, Zhang Y, Li P, Wang J, Qiu M (2015). Alteration of brain functional networks in early-stage Parkinson's disease: a resting-state fMRI study. PLoS ONE.

[CR60] Seidlitz J, Vasa F, Shinn M, Romero-Garcia R, Whitaker KJ, Vertes PE, Wagstyl K, Kirkpatrick Reardon P, Clasen L, Liu S, Messinger A, Leopold DA, Fonagy P, Dolan RJ, Jones PB, Goodyer IM, Consortium N, Raznahan A, Bullmore ET (2018) Morphometric similarity networks detect microscale cortical organization and predict inter-individual cognitive variation. Neuron 97(1):231–247. 10.1016/j.neuron.2017.11.039 10.1016/j.neuron.2017.11.039 PMC5763517 29276055

[CR61] Shah A, Lenka A, Saini J, Wagle S, Naduthota RM, Yadav R, Pal PK, Ingalhalikar M (2017). Altered brain wiring in Parkinson's disease: a structural connectome-based analysis. Brain Connect.

[CR62] Sporns O, Tononi G, Kotter R (2005). The human connectome: a structural description of the human brain. PLoS Comput Biol.

[CR63] Strogatz SH (2001). Exploring complex networks. Nature.

[CR92] Sun H, Chen Y, Huang Q, Lui S, Huang X, Shi Y, Xu X, Sweeney JA, Gong Q (2018). Psychoradiologic utility of MR imaging for diagnosis of attention deficit hyperactivity disorder: a radiomics analysis. Radiology.

[CR64] Suo X, Lei D, Li K, Chen F, Li F, Li L, Huang X, Lui S, Li L, Kemp GJ, Gong Q (2015). Disrupted brain network topology in pediatric posttraumatic stress disorder: a resting-state fMRI study. Hum Brain Mapp.

[CR65] Suo X, Lei D, Li L, Li W, Dai J, Wang S, He M, Zhu H, Kemp GJ, Gong Q (2018). Psychoradiological patterns of small-world properties and a systematic review of connectome studies of patients with 6 major psychiatric disorders. J Psychiatry Neurosci.

[CR66] Suo X, Lei D, Li N, Cheng L, Chen F, Wang M, Kemp GJ, Peng R, Gong Q (2017). Functional brain connectome and its relation to Hoehn and Yahr stage in Parkinson disease. Radiology.

[CR95] Suo X, Lei D, Li W, Li L, Dai J, Wang S, Li N, Cheng L, Peng R, Kemp GJ, Gong Q (2021). Altered white matter microarchitecture in Parkinson's disease: a voxel-based meta-analysis of diffusion tensor imaging studies. Front Med.

[CR67] Tessitore A, Esposito F, Vitale C, Santangelo G, Amboni M, Russo A, Corbo D, Cirillo G, Barone P, Tedeschi G (2012). Default-mode network connectivity in cognitively unimpaired patients with Parkinson disease. Neurology.

[CR68] Tessitore A, Giordano A, De Micco R, Russo A, Tedeschi G (2014). Sensorimotor connectivity in Parkinson's disease: the role of functional neuroimaging. Front Neurol.

[CR69] Tijms BM, Series P, Willshaw DJ, Lawrie SM (2012). Similarity-based extraction of individual networks from gray matter MRI scans. Cereb Cortex.

[CR70] Tinaz S, Lauro PM, Ghosh P, Lungu C, Horovitz SG (2017). Changes in functional organization and white matter integrity in the connectome in Parkinson's disease. Neuroimage Clin.

[CR71] Tomlinson CL, Stowe R, Patel S, Rick C, Gray R, Clarke CE (2010). Systematic review of levodopa dose equivalency reporting in Parkinson's disease. Mov Disord.

[CR72] Tzourio-Mazoyer N, Landeau B, Papathanassiou D, Crivello F, Etard O, Delcroix N, Mazoyer B, Joliot M (2002). Automated anatomical labeling of activations in SPM using a macroscopic anatomical parcellation of the MNI MRI single-subject brain. Neuroimage.

[CR73] Van Essen DC (1997). A tension-based theory of morphogenesis and compact wiring in the central nervous system. Nature.

[CR74] van Wijk BC, Stam CJ, Daffertshofer A (2010). Comparing brain networks of different size and connectivity density using graph theory. PLoS ONE.

[CR75] Vancea R, Simonyan K, Petracca M, Brys M, Di Rocco A, Ghilardi MF, Inglese M (2019). Cognitive performance in mid-stage Parkinson's disease: functional connectivity under chronic antiparkinson treatment. Brain Imaging Behav.

[CR76] Wang H, Jin X, Zhang Y, Wang J (2016). Single-subject morphological brain networks: connectivity mapping, topological characterization and test-retest reliability. Brain Behav.

[CR77] Wang J, Wang X, Xia M, Liao X, Evans A, He Y (2015). GRETNA: a graph theoretical network analysis toolbox for imaging connectomics. Front Hum Neurosci.

[CR78] Watts DJ, Strogatz SH (1998). Collective dynamics of 'small-world' networks. Nature.

[CR79] Weingarten CP, Sundman MH, Hickey P, Chen NK (2015). Neuroimaging of Parkinson's disease: expanding views. Neurosci Biobehav Rev.

[CR80] Wen MC, Heng HSE, Hsu JL, Xu Z, Liew GM, Au WL, Chan LL, Tan LCS, Tan EK (2017). Structural connectome alterations in prodromal and de novo Parkinson's disease patients. Parkinsonism Relat Disord.

[CR81] Wen MC, Xu Z, Lu Z, Chan LL, Tan EK, Tan LCS (2017). Microstructural network alterations of olfactory dysfunction in newly diagnosed Parkinson's disease. Sci Rep.

[CR82] Wu Q, Gao Y, Liu AS, Xie LZ, Qian L, Yang XG (2018). Large-scale cortical volume correlation networks reveal disrupted small world patterns in Parkinson's disease. Neurosci Lett.

[CR83] Xu J, Zhang J, Zhang J, Wang Y, Zhang Y, Wang J, Li G, Hu Q, Zhang Y (2017). Abnormalities in structural covariance of cortical gyrification in Parkinson's disease. Front Neuroanat.

[CR84] Xu X, Guan X, Guo T, Zeng Q, Ye R, Wang J, Zhong J, Xuan M, Gu Q, Huang P, Pu J, Zhang B, Zhang M (2018). Brain atrophy and reorganization of structural network in Parkinson's disease with hemiparkinsonism. Front Hum Neurosci.

[CR85] Zalesky A, Fornito A, Bullmore ET (2010). Network-based statistic: identifying differences in brain networks. Neuroimage.

[CR86] Zhang D, Liu X, Chen J, Liu B (2014). Distinguishing patients with Parkinson's disease subtypes from normal controls based on functional network regional efficiencies. PLoS ONE.

[CR87] Zhang D, Wang J, Liu X, Chen J, Liu B (2015). Aberrant brain network efficiency in Parkinson's disease patients with tremor: a multi-modality study. Front Aging Neurosci.

[CR88] Zhang DL, Liu X, Chen J, Liu B, Wang JH (2015). Widespread increase of functional connectivity in Parkinson's disease with tremor: a resting-state fMRI study. Front Aging Neurosci.

[CR89] Zhang J, Wang J, Wu Q, Kuang W, Huang X, He Y, Gong Q (2011). Disrupted brain connectivity networks in drug-naive, first-episode major depressive disorder. Biol Psychiatry.

[CR90] Zhang Y, Lin L, Lin CP, Zhou Y, Chou KH, Lo CY, Su TP, Jiang T (2012). Abnormal topological organization of structural brain networks in schizophrenia. Schizophr Res.

[CR91] Zhang Y, Qiu T, Yuan X, Zhang J, Wang Y, Zhang N, Zhou C, Luo C, Zhang J (2019). Abnormal topological organization of structural covariance networks in amyotrophic lateral sclerosis. Neuroimage Clin.

