# Supplementary material for: Disrupted morphological grey matter networks in early-stage Parkinson’s disease
Source: Brain Struct Funct. 2021 Apr 7;226(5):1389–403. doi: 10.1007/s00429-020-02200-9 (PMC8096749; doi:10.1007/s00429-020-02200-9)
Supplement: Supplementary file 1 — Supplementary file1 (DOCX 846 KB) [file 429_2020_2200_MOESM1_ESM.docx]

# *Supplementary Material for Brain Structure and Function*

**Disrupted Morphological Grey Matter Networks in Early-Stage Parkinson’s Disease**

Xueling Suo^1^, Du Lei^1,2^, Nannan Li^3^, Wenbin Li^1,2^, Graham J Kemp^4^, John A. Sweeney^1,2^, Rong Peng^3^, Qiyong Gong^1,5,6*^.

# Affiliations:

^1^ Huaxi MR Research Center (HMRRC), Department of Radiology, West China Hospital of Sichuan University, Chengdu, Sichuan, China

^2^ Department of Psychiatry and Behavioral Neuroscience, University of Cincinnati, Cincinnati, Ohio, USA

^3^ Department of Neurology, West China Hospital of Sichuan University, Chengdu, Sichuan, China

^4^ Liverpool Magnetic Resonance Imaging Centre (LiMRIC) and Institute of Life Course and Medical Sciences, University of Liverpool, Liverpool, United Kingdom

^5^ Research Unit of Psychoradiology, Chinese Academy of Medical Sciences, Chengdu, Sichuan, China

^6^ Functional and Molecular Imaging Key Laboratory of Sichuan Province, West China Hospital of Sichuan University, Chengdu, China

# Corresponding author:

Qiyong Gong MD, PhD

Email: [qiyonggong@hmrrc.org.cn](mailto:qiyonggong@hmrrc.org.cn)

# Supplementary material and methods

**1.1 Sequence Parameters of Conventional MRI Protocols**

Conventional MRI protocols were performed with a fast spin-echo sequence for the structural assessment, obtaining axial T2-weighted and ﬂuid-attenuated inversion recovery images. The scanning parameters were: (a) T2-weighted imaging: repetition time (TR) 4000 ms; echo time (TE) 93 ms; flip angle 120°; 21 axial sections; 5 mm section thickness; 230 × 320 matrix; field of view (FOV) 220 × 220 mm^2^; voxel size 0.8 × 0.7 ×5.0 mm^3^; (b) fluid-attenuated inversion recovery imaging: TR 6000 ms; TE 93 ms; flip angle 130°; 21 axial sections; 5 mm section thickness; matrix 198 × 256; FOV 220 × 220 mm^2^; voxel size 1.0 × 0.9 × 5.0 mm^3^.

# Supplementary Figures and Tables

## Supplementary Figures

**
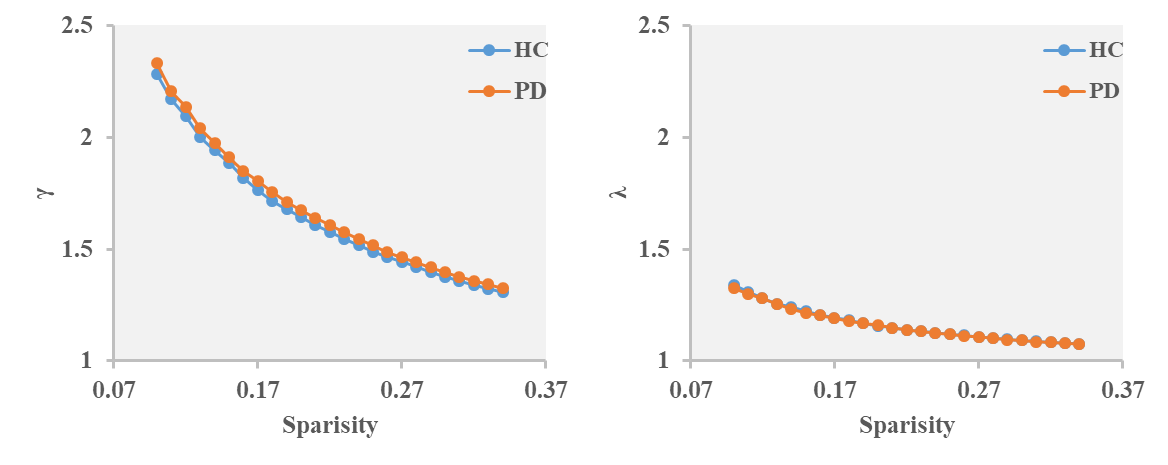
**

### Fig S1. Graphs showing that in the defined sparsity threshold range, both the early-stage PD and HC groups exhibit γ substantially larger than 1 and λ approximately equal to 1, which are the typical features of small-world topology. Abbreviations: PD, Parkinson’s disease; HC, healthy controls; γ, normalized clustering coefficient; λ, normalized characteristic path length.

###
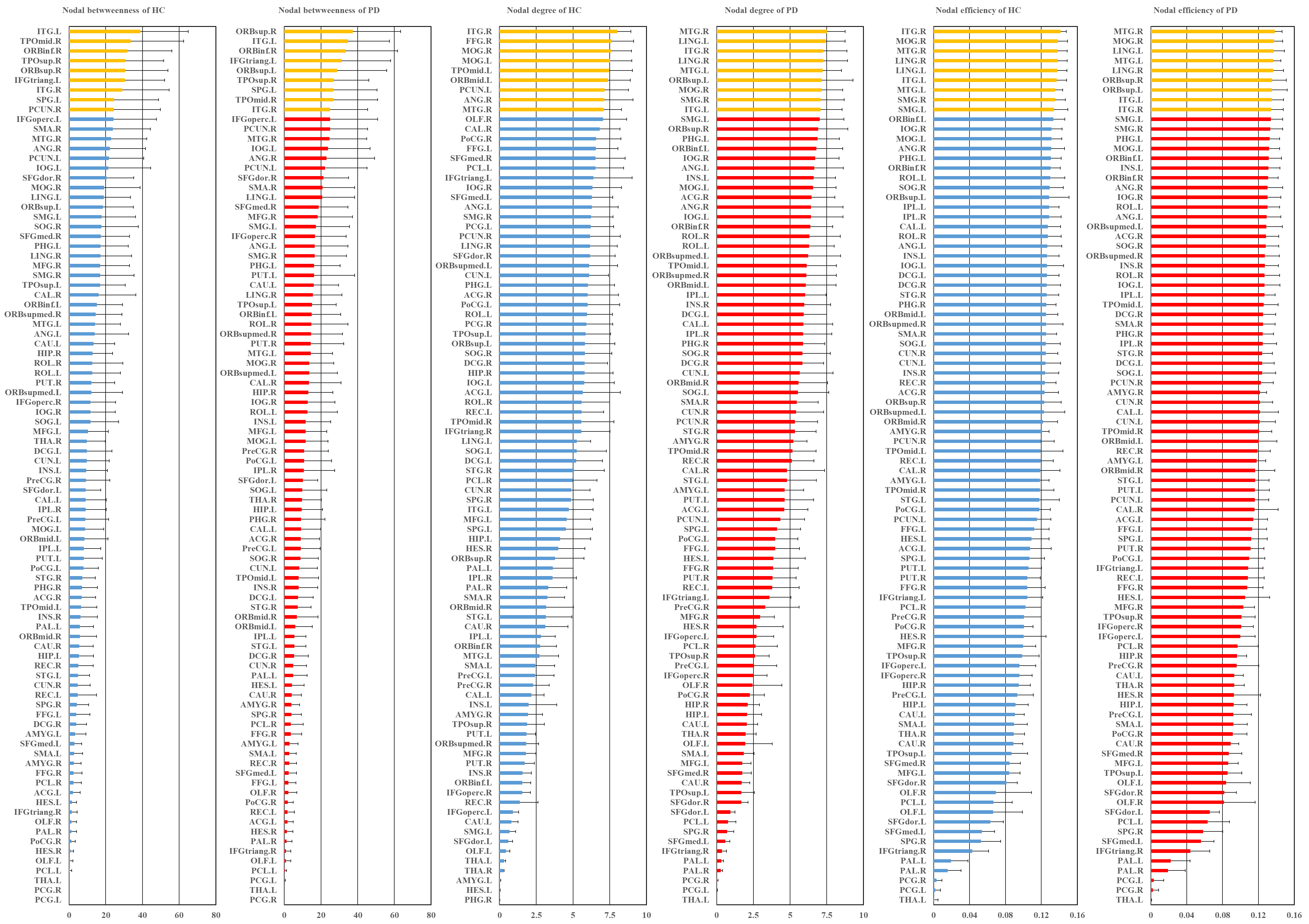
Fig S2. Nodal betweenness, nodal degree and nodal efficiency in HC and early-stage PD group. Regions with the highest centralities (top 10%) are highlighted in orange. Abbreviations: PD, Parkinson’s disease; HC, healthy controls.


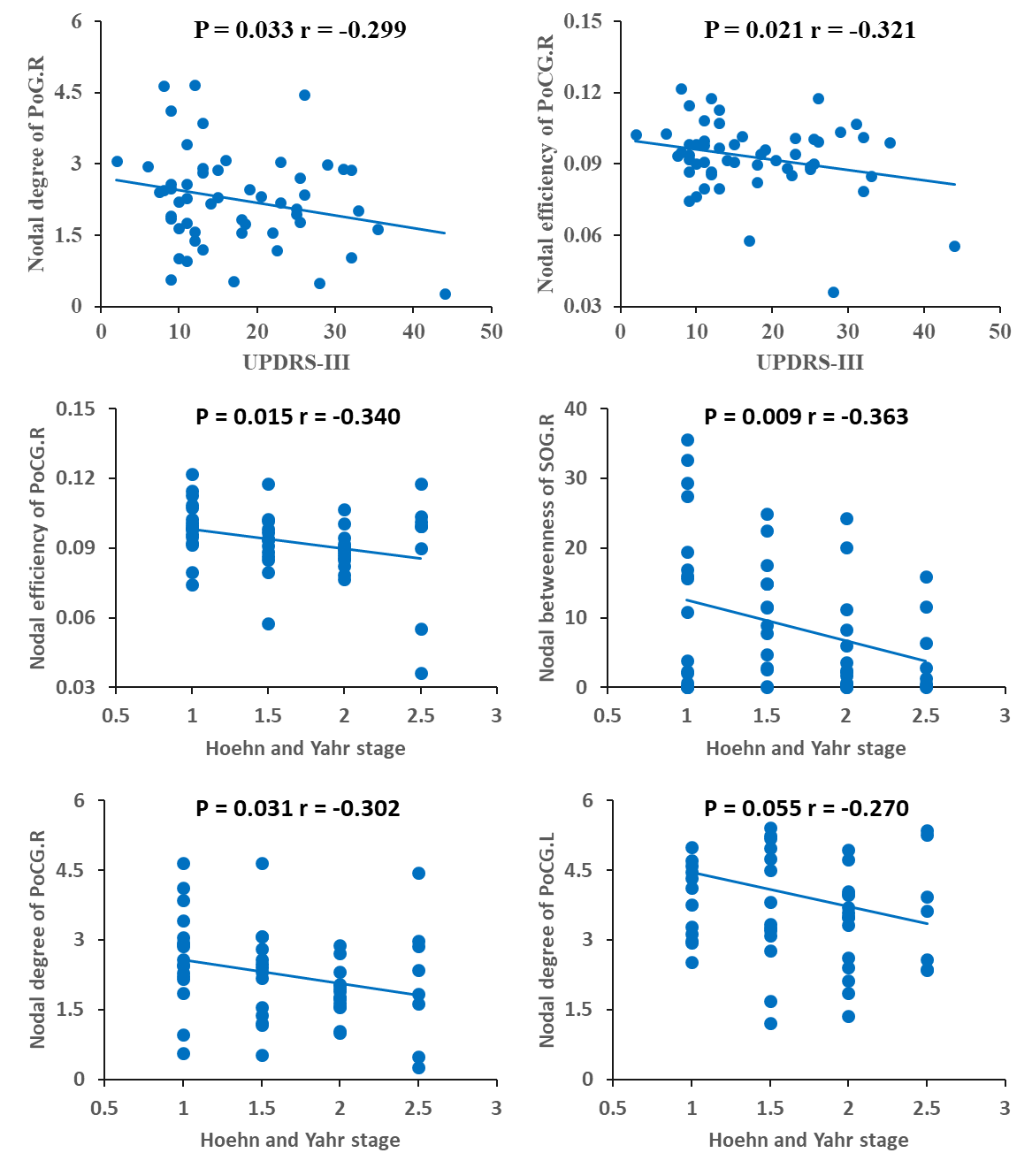


### Fig S3. Scatterplots showing correlations between the brain regions that show significant-group difference and the Hoehn and Yahr stage and UPDRS-III scores in *all* PD patients. Original P values are reported, none of which survived multiple comparisons correction at false discovery rate < 0.05. R, right; L, left; PoCG, postcentral gyrus; SOG, superior occipital gyrus; UPDRS, Unified Parkinson’s Disease Rating Scale.

###
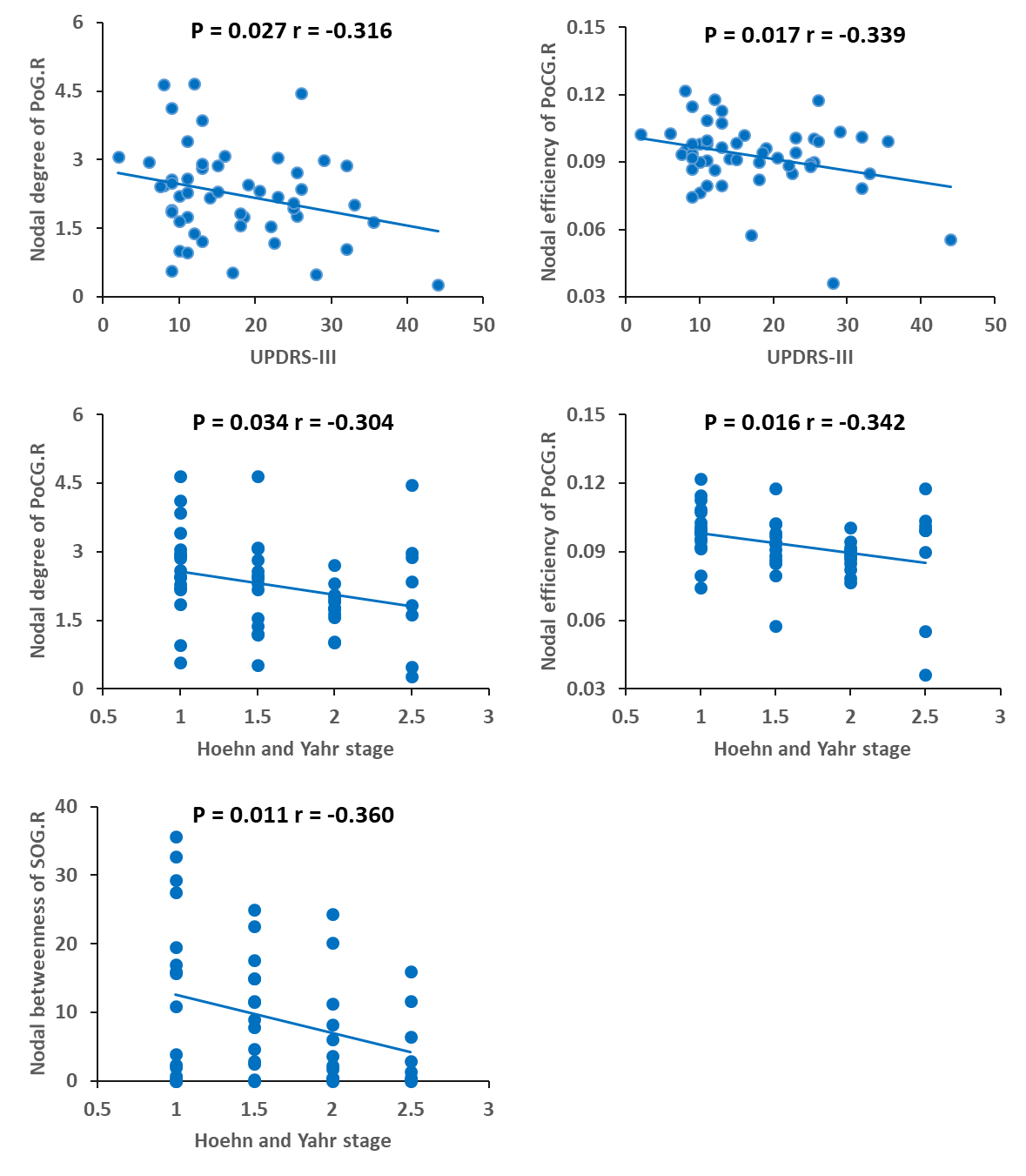
Fig S4. Scatterplots showing correlations between the brain regions that show significant between-group difference and the Hoehn and Yahr stage and UPDRS-III scores in *unilateral* PD patients. Original P values are reported, none of which survived multiple comparisons correction at false discovery rate < 0.05. R, right; PoCG, postcentral gyrus; SOG, superior occipital gyrus; UPDRS, Unified Parkinson’s Disease Rating Scale.

## Supplementary Tables

### Table S1. The AUC values of global properties in the brain grey matter networks compared between patients with early-stage Parkinson’s disease and healthy controls.

| Global properties | PD | HC | P | Cohen’s d |
| --- | --- | --- | --- | --- |
| Clustering coefficient | 0.140±0.002 | 0.139±0.002 | 0.014* | 0.574 |
| Characteristic path length | 0.556±0.016 | 0.559±0.014 | 0.100 | -0.243 |
| Normalized clustering coefficient | 0.401±0.029 | 0.394±0.027 | 0.104 | 0.243 |
| Normalized characteristic path length | 0.278±0.005 | 0.279±0.005 | 0.298 | -0.105 |
| Small-worldness | 0.344±0.028 | 0.337±0.024 | 0.098 | 0.253 |
| Local efficiency | 0.168±0.002 | 0.167±0.002 | 0.014* | 0.577 |
| Global efficiency | 0.106±0.002 | 0.106±0.002 | 0.115 | 0.234 |

Measurements presented as mean ± SD.

* P < 0.05, nonparametric permutation tests.

Abbreviations: AUC, area under the curve; PD, Parkinson’s disease; HC, healthy controls.

### Table S2. Brain regions identified as hub nodes in the morphological networks of HC and PD groups.

| Brain regions | Category | Nodal betweenness | Nodal degree | Nodal efficiency |
| --- | --- | --- | --- | --- |
| **HC group** |  |  |  |  |
| Right inferior temporal gyrus | Association | 29.078 | 8.008 | 0.141 |
| Right middle occipital gyrus | Association | 19.106 | 7.622 | 0.138 |
| Right lingual gyrus | Association | 17.189 | 7.599 | 0.138 |
| Left lingual gyrus | Association | 18.974 | 7.520 | 0.138 |
| Right middle temporal gyrus | Association | 22.988 | 7.470 | 0.138 |
| Left inferior temporal gyrus | Association | 38.926 | 7.384 | 0.137 |
| Right supramarginal gyrus | Association | 17.030 | 7.157 | 0.135 |
| Left supramarginal gyrus | Association | 17.867 | 7.125 | 0.135 |
| Left middle temporal gyrus | Association | 22.988 | 7.084 | 0.134 |
| **PD group** |  |  |  |  |
| Right middle temporal gyrus | Association | 24.670 | 7.509 | 0.138 |
| Left lingual gyrus | Association | 20.649 | 7.438 | 0.137 |
| Right inferior temporal gyrus | Association | 24.994 | 7.276 | 0.136 |
| Right lingual gyrus | Association | 15.512 | 7.268 | 0.136 |
| Left middle temporal gyrus | Association | 14.377 | 7.225 | 0.136 |
| Left superior frontal gyrus, orbital part | Paralimbic | 28.766 | 7.161 | 0.135 |
| Right middle occipital gyrus | Association | 13.659 | 7.136 | 0.136 |
| Right supramarginal gyrus | Association | 16.498 | 7.079 | 0.135 |
| Left inferior temporal gyrus | Association | 34.686 | 7.075 | 0.135 |

The cortical regions were classified as paralimbic and association ([Mesulam 1998](#_ENREF_1)).

### Table S3. Multivariate linear regression analysis in PD patients.

|  | UPDRS-III | | H&Y stage | |
| --- | --- | --- | --- | --- |
|  | F | P | F | P |
| **Global metrics** |  |  |  |  |
| Clustering coefficient | 1.283 | 0.265 | 0.912 | 0.346 |
| Local efficiency | 0.024 | 0.877 | 0.005 | 0.945 |
| **Nodal degree** |  |  |  |  |
| Right superior frontal gyrus, orbital part | 1.125 | 0.296 | 0.032 | 0.858 |
| Left gyrus rectus | 5.438 | 0.026 | 0.695 | 0.410 |
| Left postcentral gyrus | 0.212 | 0.648 | 5.599 | 0.024 |
| Right postcentral gyrus | 6.212 | 0.018 | 8.200 | 0.007 |
| Left lenticular nucleus, putamen | 3.282 | 0.079 | 8.530 | 0.006 |
| Right inferior temporal gyrus | 0.002 | 0.964 | 1.856 | 0.182 |
| **Nodal efficiency** |  |  |  |  |
| Right superior frontal gyrus, orbital part | 0.211 | 0.649 | 0.364 | 0.550 |
| Left gyrus rectus | 1.391 | 0.246 | 0.008 | 0.928 |
| Left postcentral gyrus | 3.997 | 0.053 | 2.820 | 0.102 |
| Right postcentral gyrus | 2.062 | 0.160 | 1.719 | 0.198 |
| Left lenticular nucleus, putamen | 0.400 | 0.531 | 0.370 | 0.547 |
| Right inferior temporal gyrus | 0.007 | 0.932 | 2.887 | 0.098 |
| **Nodal betweenness** |  |  |  |  |
| Right superior occipital gyrus | 6.365 | 0.016 | 2.266 | 0.141 |

Values are reported as F values followed by original P values. No one survived after correction for multiple comparisons at false discovery rate < 0.05. PD, Parkinson's disease; UPDRS, Unified PD Rating Scale; MMSE, Mini-Mental State Examination; H&Y, Hoehn and Yahr.

### Table S4. Single-subject classification in the tremor-dominant and akinetic–rigid patients with Parkinson’s disease.

| Measures | Accuracy (%) | Sensitivity ^a^ (%) | Specificity ^a^ (%) | P^b^ |
| --- | --- | --- | --- | --- |
| Grey matter networks | 53.0 | 10.0 | 96.0 | 0.134 |
| Graph-based metrics | 67.0 | 50.0 | 84.0 | **0.031** |

^a^ Sensitivity and specificity were computed considering the tremor-dominant PD as the positive class comparing with akinetic–rigid PD.

^b^ Statistical significance was estimated using the permutation method (1,000 permutations).

Abbreviations: PD, Parkinson’s disease.

### Table S5. Top 20 most relevant brain regions for the classification analysis of tremor-dominant and akinetic–rigid patients with Parkinson’s disease.

| No. | Regions | Abbreviations |
| --- | --- | --- |
| 1 | Left superior frontal gyrus, dorsolateral | SFGdor.L |
| 2 | Left supplementary motor area | SMA.L |
| 3 | Right inferior occipital gyrus | IOG.R |
| 4 | Left middle frontal gyrus, orbital part | ORBmid.L |
| 5 | Left lingual gyrus | LING.L |
| 6 | Right inferior frontal gyrus, orbital part | ORBinf.R |
| 7 | Left inferior temporal gyrus | ITG.L |
| 8 | Right cuneus | CUN.R |
| 9 | Right middle frontal gyrus, orbital part | ORBmid.R |
| 10 | Right lingual gyrus | LING.R |
| 11 | Left superior frontal gyrus, medial orbital | ORBsupmed.L |
| 12 | Right middle occipital gyrus | MOG.R |
| 13 | Left gyrus rectus | REC.L |
| 14 | Left inferior occipital gyrus | IOG.L |
| 15 | Left olfactory cortex | OLF.L |
| 16 | Left inferior frontal gyrus, triangular part | IFGtriang.L |
| 17 | Right insula | INS.R |
| 18 | Left rolandic operculum | ROL.L |
| 19 | Left temporal pole: middle temporal gyrus | TPOmid.L |
| 20 | Left amygdala | AMYG.L |

All the brain regions are from automated anatomical labelling atlas (Tzourio-Mazoyer et al. 2002).

# Supplementary references

Mesulam MM (1998) From sensation to cognition. Brain 121 ( Pt 6):1013-1052. doi:10.1093/brain/121.6.1013
